# Protocol for the visualization of bacteria in the tick gut using whole-mount fluorescence *in situ* hybridization

**DOI:** 10.1016/j.xpro.2025.103814

**Published:** 2025-05-06

**Authors:** Adnan Hodžić, Martin Kunert, David Berry

**Affiliations:** 1 Centre for Microbiology and Environmental Systems Science, Department of Microbiology and Ecosystem Science, Division of Microbial Ecology, University of Vienna, 1030 Vienna, Austria; 2 Joint Microbiome Facility of the Medical University of Vienna and the University of Vienna, 1030 Vienna, Austria

**Keywords:** Microscopy, Model Organisms, Special Issue, Protocols in Entomology

## Abstract

The tick gut harbors a relatively diverse microbial community that includes commensal, beneficial, and pathogenic bacterial species. Here, we present a protocol for the visualization of bacteria in the tick gut using whole-mount fluorescence *in situ* hybridization. We describe steps for tick dissection, tissue fixation, hybridization, washing, and mounting. We then detail procedures for microscopy imaging of the whole-mounted samples. Although the procedure is designed for gut tissue samples, it can be readily modified for use with other tissue types.

For complete details on the use and execution of this protocol, please refer to Hodžić et al.^1^

## Highlights

• Steps for dissecting ticks and preserving tick tissues
• Guidelines for tissue hybridization using double-labeled probes (DOPE-FISH)
• Instructions for washing and mounting tissue samples
• Procedures for microscopy imaging of whole-mounted samples

## Abstract (editor highlights)

Publisher’s note: Undertaking any experimental protocol requires adherence to local institutional guidelines for laboratory safety and ethics.

## Graphical abstract

**Figure:**
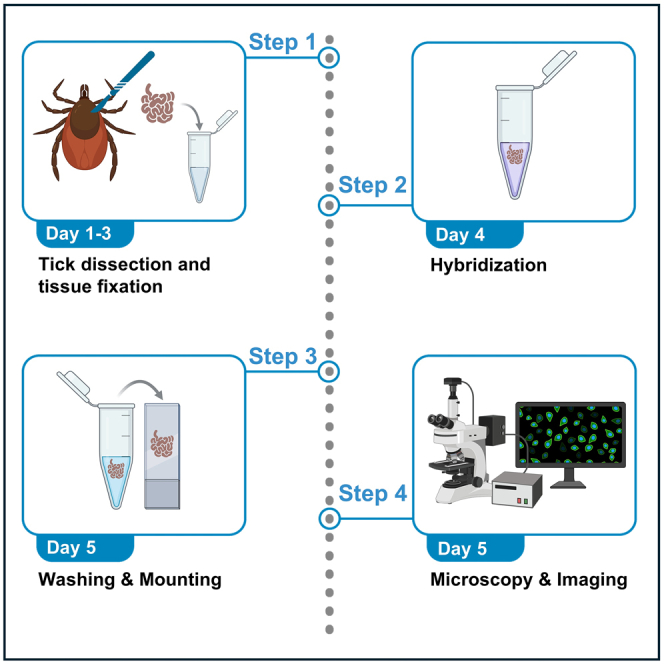

## Before you begin

Ribosomal RNA (rRNA)-targeted fluorescence *in situ* hybridization (FISH) is a powerful and versatile molecular technique extensively used for the identification, visualization, and quantification of microorganisms across a diverse range of sample types. This technique proves invaluable for investigating structural, functional, and spatial aspects of phylogenetically diverse microbial lineages, including those that cannot be cultured in laboratory settings.^2^ The protocol outlined below describes the specific steps for visualizing bacteria residing in the gut of *Ixodes ricinus* ticks, but it can also be used for the detection of bacteria in other tissues and tick species. Before you begin, ensure that all probes, reagents, chemicals, and instruments are prepared and ready for use. The exact procedures for preparing each solution can be found in the “materials and equipment” section.

### Institutional permissions

The Animals (Scientific Procedures) Act 1986 does not classify arthropods, including ticks, as animals within its scope. Consequently, research or procedures involving ticks do not require the specific ethical approvals mandated by this legislation for other animal subjects. However, for work involving field-collected ticks that potentially carry human pathogens, or for studies using animals or artificial feeding techniques for tick infection, relevant institutional permission must be obtained. Additionally, appropriate safety procedures must be implemented to prevent tick escape and minimize the risk of accidental tick exposure and bites (e.g. using tick chambers, biosafety cabinets, and sticky mats).

### Oligonucleotide probes and fluorophores

**Timing: 1–3 h**

1. First, consider using a FISH probe that has been previously published and validated. Additionally, refer to the open-source databases such as SILVA^3^ and probeBase^4^ for rRNA-targeted oligonucleotide probes.
2. To design specific probes, use the "Probe Design" and "Probe Match" functions in the ARB software package^5^ and the SILVA NR99 (release 128) 16S rRNA sequence database.^2^^,^^3^

**Table 1:** Oligonucleotide probes, their specificity and the spectral properties of the fluorophores used for labeling

Probe	Probe sequence (5′-3′)	Target organisms	Fluorophore	Excitation peak	Excitation range (nm)	Emission peak	Emission range (nm)
Borr4	CCAACACCTCACAGCACGAGC	*Borrelia* spp.	Cy3	554 nm	542–566	568 nm	578–610
EUB338-I	GCTGCCTCCCGTAGGAGT	Most Bacteria	Cy5	649 nm	622–654	667 nm	666–724
EUB338-II	GCAGCCACCCGTAGGTGT	Planctomycetales	Cy5	649 nm	622–654	667 nm	666–724
EUB338-III	GCTGCCACCCGTAGGTGT	Verrucomicrobiales	Cy5	649 nm	622–654	667 nm	666–724

The filter cube used (DFT51010) with the corresponding excitation and emission range for Cy3 and Cy5.

Note: Each probe is labeled at both its 5′ and 3′ ends. In some cases, we used EUB338 probe mix labeled with Cy3 fluorophore (see Figures 3 and 4).

**CRITICAL:** For simultaneous detection of different bacterial taxa, the oligonucleotide probes should be labeled with fluorophores that have distinct spectral properties. However, the number of simultaneously applicable fluorophores depends on the microscope filter sets. In this protocol, we used oligonucleotide probes double-labeled with sulfoindocyanine dyes Cy3 and Cy5 at their 5′ and 3′ ends (DOPE-FISH) (Table 1). The probes should be diluted to a 100 pmol/μL concentration in nuclease-free water and stored at −20°C. The probe concentration in the working solution should be 5 pmol/μL.

## Key resources table

**Table:** 

REAGENT or RESOURCE	SOURCE	IDENTIFIER
**Chemicals, peptides, and recombinant proteins**		

Formaldehyde solution 37%	Carl Roth	Cat# 4979.1
Sodium chloride ≥ 99.5%, p.a. (NaCl)	Carl Roth	Cat# 3957.1
Tris hydrochloride ≥ 99%, p.a. (Tris-HCl)	Carl Roth	Cat# 9090.2
Dextran-sulfate sodium salt from *Leuconostoc* spp.	Sigma-Aldrich	Cat# D8906
Blocking reagent	Roche	Cat# 11096176001
Sodium dodecyl sulfate (SDS) ≥ 99%	Carl Roth	Cat# 4360.2
Formamide deionized	Carl Roth	Cat# P040.1
Ethylenediaminetetraacetate (EDTA) disodium salt dihydrate ≥ 99%, p.a.	Carl Roth	Cat# 8043.2
Potassium chloride for molecular biology, ≥99.0%, p.a. (KCl)	Sigma-Aldrich	Cat# P9541
di-sodium hydrogen phosphate dihydrate ≥ 99%, p.a. (Na_2_HPO_4_ · 2H_2_O)	Carl Roth	Cat# 4984.1
Potassium dihydrogen phosphate ≥ 99% (KH_2_PO_4_)	Carl Roth	Cat# 3904.2
44',6-diamidino-2-phenylindole (DAPI)	Roche	Cat# 10236276001
Ethanol		
Milli-Q water	N/A	N/A
BioScience Grade, DEPC treated, sterile, nuclease-free water	Carl Roth	Cat# T143.3

**Experimental models: Organisms/strains**		

*Ixodes ricinus* females	Wild-caught, lab colony	N/A

**Oligonucleotides**		

EUB338 I: GCTGCCTCCCGTAGGAGT	Biomers.net	Amann et al.^6^
EUB338 II: GCAGCCACCCGTAGGTGT	Biomers.net	Daims et al.^7^
EUB338 III: GCTGCCACCCGTAGGTGT	Biomers.net	Daims et al.^7^
Borr4: CCAACACCTCACAGCACGAGC	Biomers.net	Hammer et al.^8^

**Software and algorithms**		

Leica Application Suite LAS X	Leica Microsystems	Version# 3.7.6.25997
ARB	arb-silva.de	Ludwig et al., 2004^5^

**Other**		

Leica DMi8 Thunder microscope	Leica Microsystems	Cat# 53743
Objective HC PL APO 20x/0,75 CS2	Leica Microsystems	Cat# 11506517
Objective HC PL FLUOTAR 63x/1.10 IMM CORR PH3	Leica Microsystems	Cat# 11506418
Objective HC PL APO 100x/1,40 OIL CS2	Leica Microsystems	Cat# 11506372
Leica DFC9000 GT camera	Leica Microsystems	N/A
Olympus SZ61 stereomicroscope	Olympus SZ61	Cat# SZ61-RT
LED high-power spot	Photonic	Cat# 619-30-024.99
Hybridization incubator	Memmert	Cat# UM 400
Water bath	LAUDA	Cat# L002901
Nalgene polypropylene floating microtube racks	Thermo Scientific	Cat# 5974-0404
Vortex-Genie 2	Scientific Industries	Cat# SI-0236
MiniSpin centrifuge	Eppendorf	Cat# 5452000010
Microscope slides, black coated with reaction fields	VWR/Marienfeld	Cat# MARI1216690
CitiFluor AF1, mounting medium	Scienceservices	Cat# E17970-25
Coverslips 24 × 60 mm	Marienfeld	Cat# 0102242
Microscope glass slides	Thermo Scientific	Cat# 15998086
Super glue (UHU Sekundenkleber pipette)	UHU GmbH & Co	N/A
Surgical blades no. 11	Fisher Scientific	Cat# 12697896
Fine-tipped forceps (12 cm)	Medizinische Instrumente May	Cat# PI-0039
Safe-Lock tubes (1.5 mL)	Eppendorf	Cat# 0030120086
Reaction tubes (5 mL)	Eppendorf	Cat# 0030119401

## Materials and equipment

**Table:** 70% ethanol

Reagent	Final concentration	Amount
Ethanol	70%	70 mL
Milli-Q water	N/A	30 mL

***Note:*** Store at 20°C – 22°C in a properly sealed bottle for up to 6 months.

**Table:** 10x phosphate-buffered saline (PBS)

Reagent	Final concentration	Amount
NaCl	1.37 M	80 g
KCl	26.8 mM	2 g
Na_2_HPO_4_ · 2H_2_O	101 mM	18 g
KH_2_PO_4_	17.6 mM	2.4 g
Milli-Q water	N/A	1,000 mL

***Note:*** Adjust the pH of the solution to 7.4. Autoclave the solution at 121°C for 20 min. Store at 20°C – 22°C for up to 1 year. To prepare a 1x PBS working solution, mix 10 mL of the 10x PBS stock solution with 90 mL of Milli-Q water and keep at 20°C – 22°C for 1 month.

***Alternatives:*** Na_2_HPO_4_ can be used instead of Na_2_HPO_4_ · 2H_2_O, but the difference in molecular weight must be taken into consideration.

**Table:** 4% formaldehyde solution

Reagent	Final concentration	Amount
37% Formaldehyde	4%	108.1 μL
Milli-Q water	N/A	891.9 μL

**CRITICAL:** Formaldehyde is a toxic and carcinogenic chemical. Always handle in a fume hood while wearing protective gloves and a lab coat. Dispose of it according to the institutional safety regulations.

***Note:*** Aliquot and store at −20°C for up to 4 months, or at 4°C for no more than 1 week.

**Table:** 5 M NaCl

Reagent	Final concentration	Amount
NaCl	5 M	891.9 μL
Milli-Q water	N/A	108.1 μL

***Note:*** Autoclave the solution at 121°C for 20 min and store at 20°C – 22°C for up to 4 weeks.

**Table:** 1 M Tris-HCl

Reagent	Final concentration	Amount
Tris-HCl	1 M	121.1 g
Milli-Q water	N/A	1,000 mL

***Note:*** Adjust the pH to 8. Autoclave the solution at 121°C for 20 min and store at 20°C – 22°C for up to 1 year.

**Table:** 0.5 M EDTA

Reagent	Final concentration	Amount
EDTA	0.5 M	186.1 g
Milli-Q water	N/A	1,000 mL

**CRITICAL:** Adjust the pH of the solution to 8. EDTA is difficult to dissolve in water with a pH value below 8. EDTA is a chelating agent that binds divalent ions and enhances the stabilizing effect of sodium chloride in the wash buffer.

***Note:*** Autoclave the solution at 121°C for 20 min and store at 20°C – 22°C for up to 6 months.

**Table:** 10% SDS

Reagent	Final concentration	Amount
SDS	10%	10 g
Milli-Q water	N/A	100 mL

**CRITICAL:** SDS is a hazardous chemical. Handle in a fume hood. Dispose of it according to the institutional safety regulations.

***Note:*** Make aliquots and store at 20°C – 22°C for up to 6 months.

**Table:** Hybridization buffer

Reagent	Final concentration	Amount
5 M NaCl	900 mM	1,800 μL
1 M Tris-HCl	20 mM	200 μL
Dextran-sulfate sodium salt from *Leuconostoc* spp.	N/A	1 g
Blocking reagent	N/A	1,000 μL
Formamide	20%	2,000 μL
10% SDS	0.01%	10 μL
Milli-Q water	N/A	5,000 μL

**CRITICAL:** Formamide is toxic and teratogenic. Handle it in a fume hood. Dispose of it according to the institutional safety regulations.

***Note:*** Always add SDS last to prevent possible precipitation. Store the hybridization buffer in aliquots at −20°C for up to 1 year. The concentration of formamide can vary depending on the probes used. The optimal concentration for the oligonucleotide probes used in this protocol is 20%.

**Table:** Washing buffer

Reagent	Final concentration	Amount
5 M NaCl	215 mM	43 μL
1 M Tris-HCl	20 mM	20 μL
0.5 M EDTA	5 mM	10 μL
10% SDS	0.01%	1 μL
Milli-Q water	N/A	926 μL

**CRITICAL:** Work under a fume hood. Dispose of it according to the institutional safety regulations.

***Note:*** Washing buffer should be prepared fresh right before each application to avoid precipitation.

**Table:** DAPI

Reagent	Final concentration	Amount
DAPI	10 μg/mL	10 μL
1x PBS	N/A	9,990 μL

**CRITICAL:** DAPI is a harmful substance. Always handle it in a fume hood and under dark conditions. Dispose of it according to the institutional safety regulations. Filter 1x PBS through a 0.2 μm syringe filter.

***Note:*** Store the working solution in aliquots at 4°C, protected from light, for a maximum of 6 months.

## Step-by-step method details

### Tick dissection and tissue fixation

**Timing: 10–15 min per tick for dissection, 3 days for tissue fixation**

This section outlines the procedure for dissecting female ticks, extracting their guts, and preserving the tissue samples.

**Figure 1:**
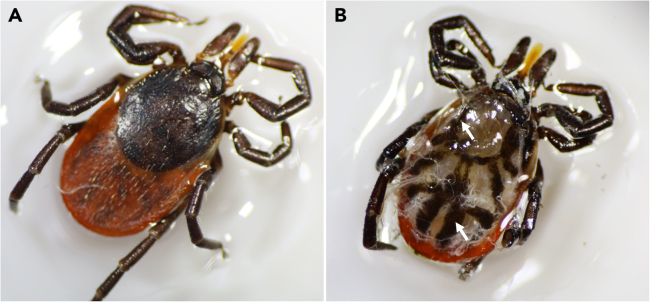
Tick dissection (A) An intact female *I*. *ricinus* tick glued on a glass slide (*dorsal view*). (B) The tick gut tissue within the body cavity after removal of the dorsal cuticular surface (*arrow*). Internal organs are covered with sterile 1x PBS to prevent tissue desiccation.
4. Under the stereomicroscope, remove the scutum/alloscutum by cutting along the edge of the tick’s body with surgical blades.
  a. Using forceps in one hand, lift the dorsal surface and cut the adhering muscles and connective tissue with the scalpel blade.
  b. Cut off the dorsal surface of the tick body and remove it.
  c. Cover the open tick cavity with sterile 1x PBS to prevent the desiccation of the internal organ tissues (Figure 1B).
  d. Remove the tracheal tubes using fine-tipped forceps to expose the internal organs.
  e. Carefully extract the gut tissue from the body and transfer it to a labeled 1.5 mL microcentrifuge tube containing 200 μL of 4% formaldehyde solution.
5. Preserve the tissue in 4% formaldehyde solution at 4°C for 3 days.

***Note:*** Up to 5 unfed female ticks may be adhered to a glass slide. Individual or pooled guts can be preserved in a single tube. Other tissues (e.g. ovaries, salivary glands) should be preserved in a similar manner. Ensure that the tissues are fully submerged in the 4% formaldehyde. The remaining tick body parts should be disposed of as biohazardous waste.

1. Before dissection, wash the ticks in a 5 mL tube with 70% ethanol for 1 minute, then rinse them in three successive baths of sterile Milli-Q water to remove surface contaminants. Ticks from the same group can be pooled together.
2. Remove excess moisture from the tick’s surface by gently patting it with filter paper.
3. Affix the ticks to a glass slide using super glue (Figure 1A).

***Alternatives:*** The 4% paraformaldehyde solution is also suitable for tissue fixation. Whole ticks can be fixed prior to dissection. This approach is especially advantageous when working with ticks that are partially or fully engorged, as the fixation strengthens the tissue, making it more resilient during the dissection process.

***Optional:*** Other dissection techniques can also be used for nymphal and female ticks.^9^^,^^10^

### Oligonucleotide probe hybridization

**Timing: 16–18 h**

This section describes the steps for hybridizing DOPE-FISH oligonucleotide probes to the target sequences of specific bacterial taxa.

***Note:*** Begin this step in the afternoon (around 3 p.m.). Set the incubator temperature to 46°C and preheat the hybridization buffer. Perform the following steps under a fume hood.

6. Using a pipette, gently aspirate as much of the fixative as possible, taking care to avoid touching or damaging the tissue.
7. Wash the tissue with 1 mL of 1x PBS.
8. Repeat step 6.

***Note:*** Discard the fixative and the PBS wash according to the institutional safety regulations. Excess residual solution may alter the concentration of the hybridization buffer, potentially compromising the results of the experiment.

9. Add 20 μL of the working oligonucleotide probe solutions to 200 μL of hybridization buffer preheated to 46°C and mix thoroughly (0.5 pmol/μL final probe concentration).
10. Add the hybridization buffer to the microcentrifuge tube containing the gut tissue.
11. Incubate the sample at 46°C overnight (16 – 18 h) in an incubator.

**CRITICAL:** Avoid exposing the probes or the hybridization buffer containing the probes to light.

***Note:*** Make sure that the tick tissue is fully submerged in the hybridization buffer. The optimal signal-to-noise ratio in the microscopy images is achieved when the hybridization time is between 16 to 18 h.

### Washing, counterstaining, and tissue mounting

**Timing: 1 h**

The steps below outline the process of removing unbound probes from the samples, followed by DAPI staining, and sample mounting for microscopy.

***Note:*** Preheat the water bath and washing buffer to 48°C. Prepare fresh Milli-Q water and cool it on ice. Perform all steps under a fume hood.

12. Carefully remove as much of the hybridization buffer as possible and dispose of it according to the institutional safety regulations.

**CRITICAL:** Heated formamide is very volatile; therefore, open the microcentrifuge tube only under a fume hood.

13. Carefully add 1 mL of preheated washing buffer to the sample in the microcentrifuge tube.
14. Put the tube in the floating rack and submerge it in the 48°C water bath for 30 min.

***Note:*** To maximize the washing effect, gently invert the tube every 10 min, making sure that the gut tissue is completely submerged in the buffer afterward.

15. Remove the washing buffer under a fume hood and dispose of it according to the institutional safety regulations.
16. Add 1 mL of 1x PBS to the sample and incubate in the dark at 20°C – 22°C for 15 min.

***Note:*** Thoroughly remove the excess liquid to maintain the proper concentration of DAPI solution.

17. Add 200 μL of DAPI solution (10 μg/mL) to the sample and incubate for 8 min at 20°C – 22°C in the dark.
  a. Discard the DAPI solution and rinse the sample with 1 mL of ice-cold Milli-Q water.
  b. Remove the water under a fume hood and dispose of it according to the institutional safety regulations.

***Note:*** The use of DAPI as a nuclear stain for host cells enhances the ability to precisely locate bacteria within the structural context of tick tissues.

18. Using scissors, trim the end of a 1,000 μL pipette tip to widen its opening and carefully aspirate the gut tissue along with the remaining liquid from the bottom of the tube.
  a. Transfer the tissue to a reaction well on a glass slide.
  b. Remove excess water from the reaction well with a 20 μL pipette.
  c. Place a drop of Citifluor AF1 antifade reagent between each reaction well on a glass slide and cover with a 24x60 mm coverslip. Avoid air bubbles!

***Note:*** To obtain high-quality microscopy images, the tissue must be completely embedded in mounting medium.

***Optional:*** DAPI can be added to the antifade mounting medium during slide preparation.

**Pause Point:** Mounted slides can be stored for up to 24 h at 4°C before microscopy.

### Microscopy and imaging

**Timing: 30–45 min per sample**

This section describes the microscopy settings and steps used for imaging whole-mounted FISH samples.

***Note:*** Due to the size and complexity of the tissue, it may sometimes be necessary to combine adjacent individual images into one (tile scan) or superimpose them using a *z*-stack with maximum projection. In the following steps, we will outline all the necessary procedures to achieve these results.

***Note:*** We utilized the Leica DMi8 Thunder microscope along with LAS X software. However, the individual steps are articulated in a manner that enables reproducible results with other microscope models.

***Note:*** When comparing signal intensity between samples, maintain consistent laser and exposure settings across all channels for each sample. This ensures accurate comparisons. However, if your goal is to obtain the highest quality image for each sample individually, you may adjust these parameters as needed. This flexibility is useful because tissue density and hybridization intensity can vary from one sample to another.

**CRITICAL:** It is recommended to avoid excessive laser power and exposure, as the fluorophores are sensitive to light and can bleach.

19. Start the microscope and set all channels to the specific excitation and emission wavelengths of the fluorophore you are using.
20. Add the immersion medium appropriate for the objective onto the lens and then position the slide with the mounted specimen on the microscope stage.
21. Turn on one of the fluorescence channels and locate the focal plane using the eyepiece lenses.

***Note:*** We use DAPI to locate the focal plane because its signal is often stronger than that of the fluorophores.

22. Inspect the hybridization signals to determine the appropriate laser power.
  a. Use an objective with lower magnification to obtain an overview of your sample. If you are already familiar with the structure of the sample, you can opt for a higher magnification.

***Note:*** In our experiment, we used the Leica DMi8 Thunder microscope with a 20x, 63x and 100x objective. The 20x objective in combination with bright field was used to get an overview of the location of the gut tissues. For capturing precise and detailed fluorescence images, we used the 63x and 100x objectives.

23. To ensure the success of hybridization and to avoid potential failure, always check the strength and specificity of the probe signals in all channels using the eyepiece.
24. Before acquiring an image, the following settings should be adjusted:
  a. Find a balance between the areas with specific signals and non-specific labeled tissues and the background.
  b. The signal strength of the target area should be substantially higher than that of the background.
25. Adjust the laser power and exposure time for image acquisition in live mode.

***Note:*** Many cameras offer a “binning” option that increases light sensitivity by combining several pixels.

26. Acquire the image with all set channels.
  a. Control whether all individual signals are in focus and well-matched in terms of signal intensity within the overlay.

***Note:*** If a larger section of tissue needs to be imaged, continue with the Tile scan. If the signals are on different focal planes, use a *z*-stack with maximum projection.

27. Select the tile scan option (navigator).
28. In live mode, define the outer limits of the area to be imaged.

***Note:*** The number of individual images needed is calculated based on the size of the marked area.

29. Verify if the selected area is in focus.
  a. If the points of interest are not on the same plane, define individual focus points for separate images, creating a focus map.
  b. Check if all signals in the individual channels are in focus and align well in terms of signal intensity.
30. Select the option for the individual images to overlap by 10% and be merged after capture.
31. Acquire the image and verify the quality in each individual channel as well as in the overlay.

***Note:****Z*-stack can be used with single images or in combination with the tile scan.

32. Open the *z*-stack option and start the live mode.
  a. Focus the target signals on the upper level of your sample and set the starting point for the *z*-stack.
  b. To set the end point for the *z*-stack repeat at the lower level of interest.

***Note:*** The LAS X software will calculate the number of steps needed. If necessary, they can also be set individually.

33. Check if all signals in the individual channels match in terms of signal intensity.
34. Acquire the *z*-stack.
35. Select the maximum projection to turn the *z*-stack into a single 2D image.

***Note:*** Methods such as computational clearing (Thunder) can be used to eliminate the unfocused background, thereby enhancing the visibility of the signal of interest.

36. Export all relevant images for further analysis.

## Expected outcomes

The whole-mount FISH protocol described here enables the visualization, identification, and spatial mapping of bacterial species within the tick gut. Additionally, it is useful for quantifying pathogen infection levels in naturally and experimentally infected ticks. When performed correctly, this protocol should allow researchers to observe fluorescent bacteria exclusively in the channel corresponding to the specific fluorophore used for oligonucleotide probe labeling (Table 1). Importantly, these bacterial signals should not be visible in channels associated with other excitation/emission spectra. This specificity ensures accurate identification of the target bacterial species.

**Figure 2:**
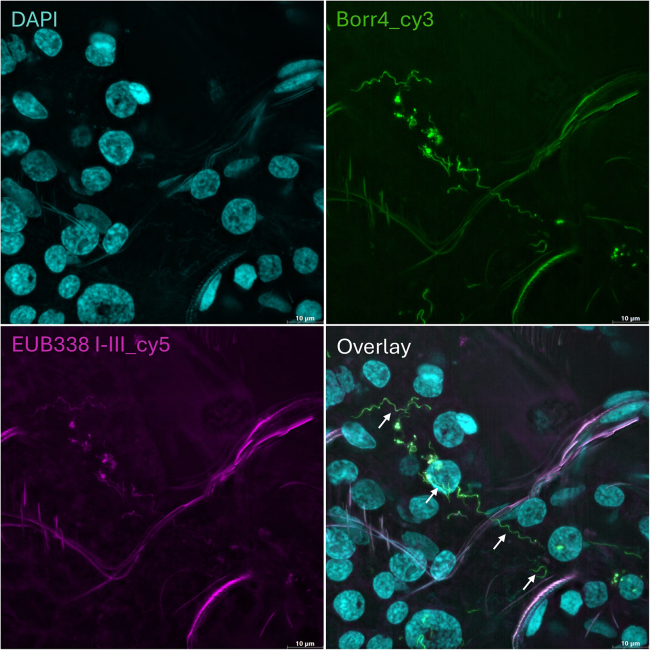
A representative overview of whole-mount FISH in *I*. *ricinus* gut *Borrelia* spirochetes are visible within the gut lumen of an unfed female tick (*arrows*). Host nuclei and bacterial cells were stained with DAPI, while Borr4 and EUB338 mix oligonucleotide probes were used simultaneously to stain the spirochetes. Scale bar: 10 μm.

**Figure 3:**
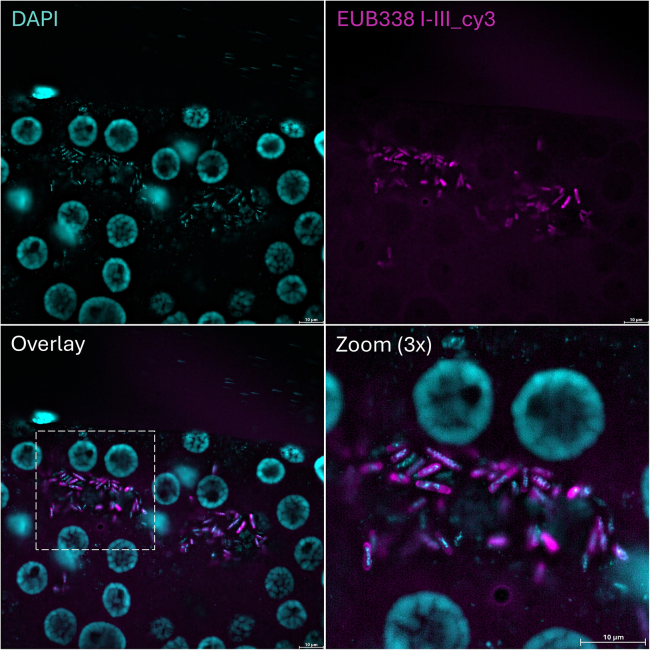
A whole-mount FISH image shows the presence of rod-shaped bacteria in the gut lumen of an unfed *I. ricinus* female tick The bacteria were stained using the EUB338 probe mix. DAPI was used to stain nuclei. Scale bar: 10 μm.

**Figure 4:**
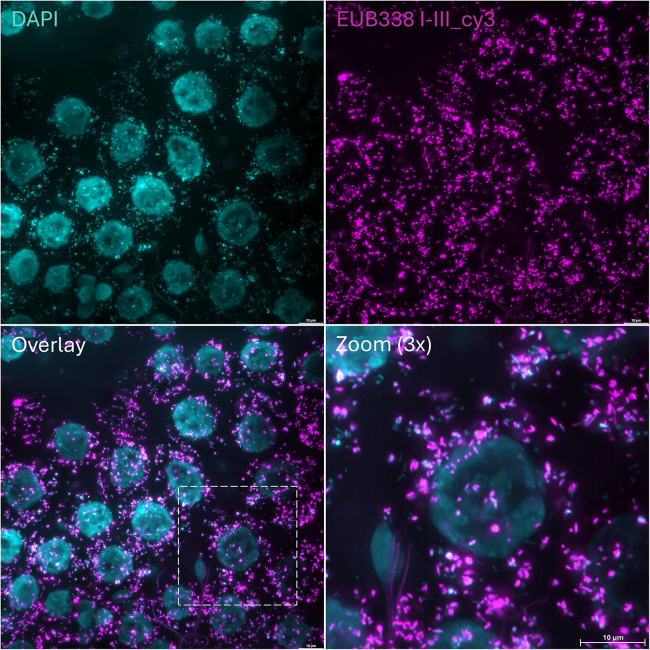
A representative image of whole-mount FISH shows intracellular bacteria that likely correspond to the *Midichloria mitochondrii* endosymbionts in the ovaries of an *I. ricinus* female tick from a lab colony The bacteria were stained with the EUB338 probe mix, while the nuclei were stained using DAPI. Scale bar: 10 μm.

The EUB338 mix, comprising EUB338-I, EUB338-II, and EUB338-III probes, enhance the detection of a wider range of bacterial groups (Table 1).^6^^,^^7^ In contrast, the Borr4 probe is specifically designed to target the rRNA gene of *Borrelia* species (Table 1).^8^ While the EUB338 mix provides a broad overview of bacterial presence, the *Borrelia* probe enables specific identification of the pathogenic bacteria. Figures 2 and 3 illustrate example outcomes. Notably, the versatility of this protocol extends beyond the tick gut tissue, as it can be applied to detect bacteria in other tick organs as well (Figure 4).

## Limitations

As with standard FISH, our protocol has limitations regarding the number of phylogenetically diverse target organisms that can be detected simultaneously due to constraints in distinguishing multiple fluorophores. The current setup allows for the simultaneous detection of DNA containing microbial and host cells, and up to three distinct rRNA targets, using DAPI, Cy3, Cy5, and FLUOS fluorescent dyes, respectively. Additionally, this method may have reduced effectiveness in detecting bacteria that are present in low abundance and in targeting closely related lineages that have similar rRNA sequences but may be functionally divergent.

## Troubleshooting

### Problem 1

No signal can be detected (related to Step 19).

### Potential solution

If this happens, ensure that the correct settings for the light source channels are used in accordance with the fluorophores. If possible, evaluate the binding efficacy of the selected probe *in vitro* using fixed samples of the target bacteria and assess its off-target binding to other bacterial species.

### Problem 2

Signal intensity is too high (related to Step 9, 11, 19, and 22).

### Potential solution

This issue can potentially be resolved by using a lower concentration of probes, shortening the hybridization time, adjusting the microscope settings, or reducing the laser intensity and the camera’s exposure time.

### Problem 3

The background noise is too strong (related to Step 11, 14, and 16).

### Potential solution

If the hybridization time is too long, the probability of probes being accidentally deposited increases. Therefore, shorten the hybridization time and/or prolong the washing step to remove non-specific binding.

### Problem 4

Strong background noise reduces the effectiveness of the EUB338 probe mix for bacterial detection in blood-fed guts (related to the “before you begin” section).

### Potential solution

**Figure 5:**
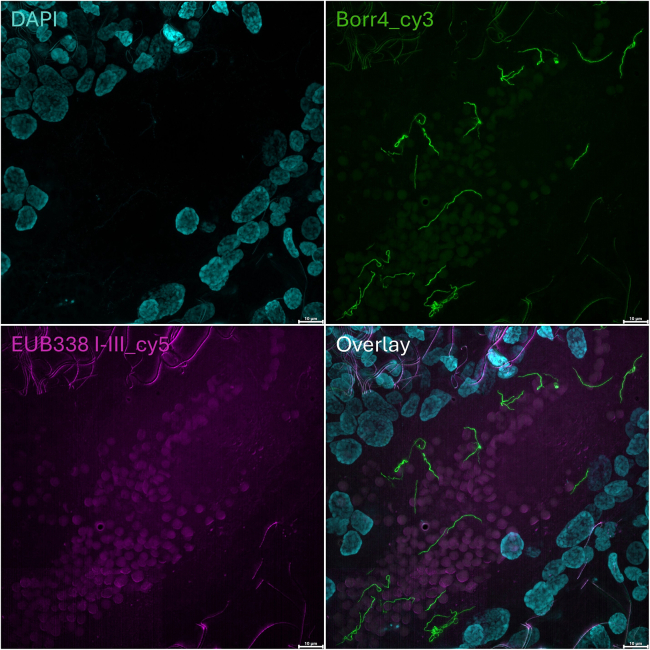
The EUB338 probe mix exhibits reduced sensitivity in detecting *Borrelia* spirochetes in the gut of blood-fed ticks compared to the *Borrelia*-specific probe The spirochetes were simultaneously stained with Borr4 and EUB338 mix oligonucleotide probes. DAPI was used to stain DNA containing host and microbial cells. Scale bar: 10 μm.

Signal-to-noise ratio in partially or fully engorged ticks can be improved by using a genus- or species-specific probe (see Figure 5). Based on our observations, probes labeled with the Cy3 fluorophore tend to produce superior results compared to those labeled with Cy5 in both unfed and blood-fed ticks.

### Problem 5

Certain tissues can have autofluorescence in the different fluorescence channels (related to the “microscopy and imaging” section).

### Potential solution

Make sure to use channels which have the lowest autofluorescence. Check that fluorescence does not originate from unspecific binding of the probes. Perform control experiments by hybridizing the tissue without probes or with a nonsense probe.

## Resource availability

### Lead contact

Further information and requests for resources and reagents should be directed to and will be fulfilled by the lead contact, Adnan Hodžić (adnan.hodzic@univie.ac.at).

### Technical contact

Technical questions on executing this protocol should be directed to and will be answered by the technical contact, Martin Kunert (martin.kunert@univie.ac.at).

### Materials availability

The above protocol does not entail the use of any new, unique reagents.

### Data and code availability

The above protocol does not entail the use of any new datasets or codes.

## Acknowledgments

This work was funded in whole or in part by the Austrian Science Fund (FWF) (grant DOI: https://doi.org/10.55776/P36130). For open access purposes, the authors have applied a CC BY public copyright license to any author-accepted manuscript version arising from this submission. The graphical abstract was created using Biorender.com.

## Author contributions

A.H.: conceptualization, resources, visualization, and writing – original draft. M.K.: data curation, investigation, visualization, and writing – review and editing. D.B.: formal analysis, supervision, and writing – review and editing.

## Declaration of interests

The authors declare no competing interests.
